# Proteomic Identification of Target Proteins of Thiodigalactoside in White Adipose Tissue from Diet-Induced Obese Rats

**DOI:** 10.3390/ijms160714441

**Published:** 2015-06-25

**Authors:** Hilal Ahmad Parray, Jong Won Yun

**Affiliations:** Department of Biotechnology, Daegu University, Kyungsan, Kyungbuk 712-714, Korea; E-Mail: hilalparray@gmail.com

**Keywords:** galectin, obesity, proteome, thiodigalactoside, white adipose tissue

## Abstract

Previously, galectin-1 (GAL1) was found to be up-regulated in obesity-prone subjects, suggesting that use of a GAL1 inhibitor could be a novel therapeutic approach for treatment of obesity. We evaluated thiodigalactoside (TDG) as a potent inhibitor of GAL1 and identified target proteins of TDG by performing comparative proteome analysis of white adipose tissue (WAT) from control and TDG-treated rats fed a high fat diet (HFD) using two dimensional gel electrophoresis (2-DE) combined with MALDI-TOF-MS. Thirty-two spots from a total of 356 matched spots showed differential expression between control and TDG-treated rats, as identified by peptide mass fingerprinting. These proteins were categorized into groups such as carbohydrate metabolism, tricarboxylic acid (TCA) cycle, signal transduction, cytoskeletal, and mitochondrial proteins based on functional analysis using Protein Annotation Through Evolutionary Relationship (PANTHER) and Database for Annotation, Visualization, Integrated Discovery (DAVID) classification. One of the most striking findings of this study was significant changes in Carbonic anhydrase 3 (CA3), Voltage-dependent anion channel 1 (VDAC1), phosphatidylethanolamine-binding protein 1 (PEBP1), annexin A2 (ANXA2) and lactate dehydrogenase A chain (LDHA) protein levels between WAT from control and TDG-treated groups. In addition, we confirmed increased expression of thermogenic proteins as well as reduced expression of lipogenic proteins in response to TDG treatment. These results suggest that TDG may effectively prevent obesity, and TDG-responsive proteins can be used as novel target proteins for obesity treatment.

## 1. Introduction

Obesity results from continuous imbalance between energy intake and energy expenditure. White adipose tissue (WAT) plays an important role in the progression of obesity and metabolic diseases. Besides release of fatty acids and metabolites, adipose tissue secretes more than 600 bioactive factors known as adipokines [1]. These adipokines modulate adipogenesis as well as adipocyte metabolism and function. Although adipose tissue was earlier considered as only an energy storage organ, it has been shown to play important roles in metabolism as well as energy homeostasis [2,3].

Among WAT proteins, galectins have been measured at higher concentrations in obese subjects [4]. More than 14 mammalian galectins with distinctive functions in various cellular processes have been identified to date [5]. Galectins play important roles in several intra- and extracellular processes, including cell–cell adhesion, intracellular vesicle transport, as well as cellular growth and apoptosis [6,7,8]. Galectins bind to acetyllactosamine found on various *N*- or *O*-linked glycans and are able to specifically interact with other ligands [9]. Among the galectin family, galectin 3 (GAL3), GAL9, and GAL12 are the most well described members in the context of obesity, diabetes, and cancer and possess different physiological roles [4,10]. GAL12 plays an important role in regulation of adipose tissue development, and its deficiency leads to increased lipolysis as well as reduced adiposity and insulin resistance [8,9]. GAL3 plays a pro-inflammatory role in adipose tissue, whereas GAL 9 has anti-inflammatory and anti-microbial activities as well as induces maturation of dendritic cells [11].

Galectin-1 (GAL1) is a prototypical galectin that possesses only a single carbohydrate recognition domain and forms symmetric homodimers based on its hydrophobic faces [12]. Although the specific roles of galectins are yet to be elucidated, GAL1 has been shown to invoke apoptosis in thymocytes as well as aggravate T cell receptor signaling [13,14]. Recently, higher expression of GAL1 has been reported in subcutaneous adipose tissue from obese subjects and diet-induced obese mice [4]. Higher concentration of GAL1 has also been reported in adipocytes during the differentiation stage [5]. Taken together, targeted inhibition of GAL1 may help prevent obesity.

Glycoconjugates such as galactose, lactose, and thiodigalactoside (TDG) as well as some peptides such as davant and anginex are known to display different binding affinities for galectin family members and act as binding inhibitors [15,16]. Among these glycoconjugates, TDG is a non-metabolizable disaccharide and known strongest inhibitor of GAL1 [17]. In order to characterize protein functions and translational modifications, separation, characterization, and identification of the proteome is essential [18,19]. Thus, two-dimensional electrophoresis (2-DE) coupled with MALDI-TOF-MS is considered to be a powerful tool for the separation of adipose tissue proteins [20,21,22]. This approach could possibly be used to identify novel biomarkers related to obesity treatment.

To address the issue of diet-induced obesity prevention by glycoconjugates, further knowledge of adipose tissue dysfunction is needed. In the present study, in an attempt to identify target proteins of TDG, we investigated differential regulation of WAT proteins in response to TDG treatment. To the best of our knowledge, this is the first proteomic study of WAT upon TDG treatment in an obese animal model.

## 2. Results

### 2.1. Thiodigalactoside (TDG) Significantly Reduces Body Weight Gain in High Fat Diet (HFD) Fed Rats

**Figure 1:**
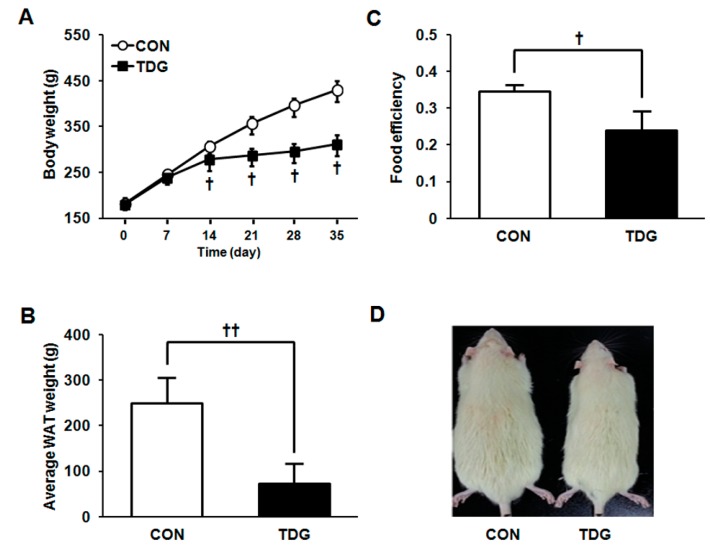
Time profiles of body weight gain (**A**) average white adipose tissue (WAT) weight (**B**) and food efficiency (**C**) between control and thiodigalactoside (TDG)-treated rats; (**D**) Image of control and TDG-treated rats. Statistical significance between control and TDG-treated groups was calculated by Student’s *t-*test, where *p*-values are ^†^ *p <* 0.05 and ^††^ *p <* 0.01.

We evaluated the anti-obesity effects of TDG in high fat diet (HFD) induced obese rats by treatment with 5 mg/kg of TDG for five weeks (once per week). As shown in Figure 1A, intraperitoneal TDG treatment dramatically reduced the body weight gain (27% as compared to HFD-fed controls). Correspondingly, WAT mass and food efficiency were significantly reduced in the TDG-treated group (Figure 1B,C). Figure 1D depicts the size difference between control and TDG-treated group. Taken together, these data suggest that TDG is a potent anti-obesity drug candidate and prompted us to perform further studies related to proteomics.

### 2.2. Proteomic Analysis of White Adipose Tissue (WAT)

**Figure 2:**
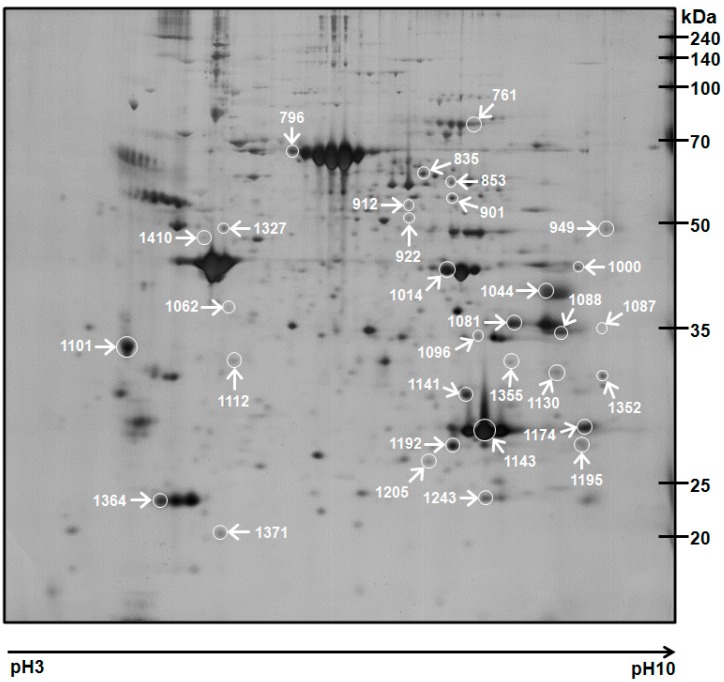
Representative silver-stained two-dimensional electrophoresis (2-DE) gel image of WAT proteome. Differentially regulated proteins are marked with white circles. Thirty-two proteins from a total of 356 matched spots showed differential expression between control and TDG-treated groups.

Pooled WAT protein samples from the two groups (control (CON) and TDG groups) were separately resolved by 2-DE using broad-range isoelectric focusing (IEF) strips. Protein spots were identified by MALDI-TOF-MS (Table 1), and database searches were performed with high confidence based on sequence coverage and high score (*p <* 0.05). A total of 356 individual matched spots ranging from 6 to 240 kDa between pH 3–10 were detected (Figure 2). Thirty-two identified proteins showed differential regulation between the CON- and TDG-treated groups. These differentially altered spots showed dramatic changes in response to TDG, suggesting that TDG may play a significant role in body weight reduction in HFD-fed obese rats.

**Table 1:** List of proteins showing differential expression between control and thiodigalactoside (TDG)-treated rats.

Spot ID	Description	Accession No. ^a^	Nominal Mass (M_r_) ^b^	Calculated pI	Fold Change	Score ^c^	Number of Peptides Matched	Sequence Coverage (%)
	Carbohydrate metabolism							
835	Phosphoglucomutase-1 (PGM1)	gi|77627971	61,637	6.14	1.97	229	24	48
853	Pyruvate kinase (PKM)	gi|16757994	58,294	6.63	2.77	159	20	43
1081	Glyceraldehyde-3-phosphate dehydrogenase (GAPDH)	gi|8393418	36,090	8.14	2.31	191	23	59
1044	Aldolase A (ALDOA)	gi|202837	39,691	8.31	1.80	222	17	64
1088	l-lactate dehydrogenase A chain (LDHA)	gi|8393706	36,712	8.45	4.22	167	15	50
1192	Triosephosphate isomerase (TPI1)	gi|117935064	27,345	6.89	2.89	237	17	72
1174	Phosphoglycerate mutase 2 (PGAM2)	gi|8393948	28,908	8.85	4.07	104	12	44
	TCA cycle							
1087	Malate dehydrogenase, mitochondrial precursor (MDH2)	gi|42476181	36,117	8.93	6.06	238	20	57
1112	Pyruvate dehydrogenase E1 component subunit beta (PDHB)	gi|56090293	39,299	6.20	2.81	92	13	31
1327	Cytochrome b-c1 complex subunit 1 (UQCRC1)	gi|51948476	53,500	5.57	2.36	151	12	47
1000	Acetyl-Coenzyme A acyltransferase 2 (ACAA2)	gi|149027152	25,270	8.22	2.29	164	15	37
1143	Carbonic anhydrase 3 (CA3)	gi|31377484	29,698	6.89	2.79	234	17	61
	Signal Transduction							
1195	GTP:AMP phosphotransferase AK3, mitochondrial (AK3)	gi|6978479	25,479	8.89	2.18	78	9	36
1243	Adenylate kinase isoenzyme 1 (AK1)	gi|61889092	21,684	7.66	4.63	177	18	39
1371	Phosphatidylethanolamine-binding protein 1 (PEBP 1)	gi|8393910	20,902	5.48	4.08	65	4	40
1014	Creatine kinase M-type (CK)	gi|6978661	43,220	6.58	5.55	257	20	58
	Cytoskeletal proteins/Mitochondrial							
1096	Annexin A2 (ANXA2)	gi|9845234	38,939	7.55	2.14	64	5	15
1130	Vdac1 protein, partial (VDAC1)	gi|38051979	32,060	8.35	3.83	309	21	72
1101	Tropomyosin alpha-1 chain (TPM1)	TPM1_RAT	32,718	4.69	6.70	95	9	42
912	Myosin-6 (MYH6)	MYH6_RAT	224,168	5.59	3.12	61	8	30
922	Inner membrane protein, mitochondrial, isoform CRA_a (IMMT)	gi|149036390	86,204	5.67	1.80	64	13	28
1355	Myozenin-1 (MYOZ 1)	gi|157819165	31,379	8.57	6.29	134	15	48
1352	LIM domain-binding protein 3 isoform 4 (LDB3)	gi|160333156	30,969	9.17	9.32	106	11	46
1364	Myosin light chain (MLC1-F)	gi|205485	20,793	4.99	7.77	94	8	49
796	Others Serum albumin precursor (ALB)	gi|158138568	70,710	6.09	1.72	239	22	39
1141	Carbonyl reductase 1 (CBR1)	gi|9506467	30,844	8.22	2.97	199	19	43
1205	Flavin reductase (BLVRB)	gi|157819619	22,194	6.29	4.57	63	4	21
761	Serotransferrin precursor (TF)	gi|61556986	78,512	7.14	3.92	194	19	27
1062	Alpha-1-macroglobulin precursor (PZP)	gi|307746876	168,388	6.46	2.81	65	11	36
949	Elongation factor 1-alpha 1 (EEF1A1)	gi|28460696	50,424	9.10	1.57	79	10	27
1410	rCG36867	gi|149050147	8002	8.65	2.83	73	4	20
901	Cysteine-sulfinate decarboxylase (CSAD)	gi|193072901	55,807	6.84	2.18	105	13	30

^a^ NCBInr/SWISS database accession number; ^b^ The nominal mass is the integer mass of the most abundant naturally occurring stable isotope of an element; ^c^ MASCOT probability-based molecular-weight search score calculated for peptide mass fingerprinting (PMF). Protein score is 10 × log(*p*), where *p* is the probability that the observed match is a random event; it is based on the NCBInr database using the MASCOT searching program as MS/MS data and protein scores >61 are significant (*p* < 0.05).

### 2.3. Differential Regulation of WAT Proteins

**Figure 3:**
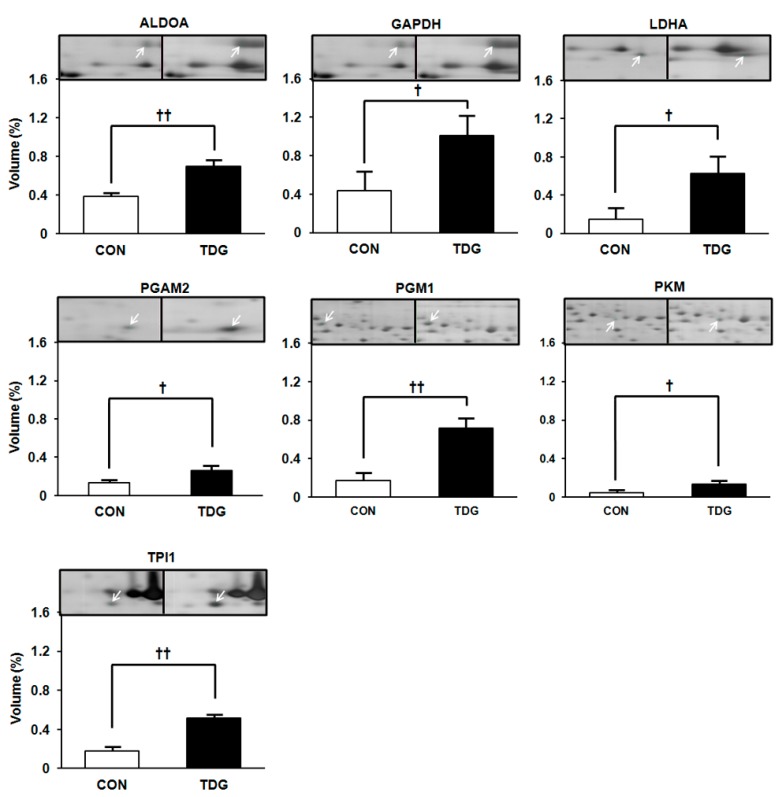
WAT proteins involved in carbohydrate metabolism showing differential regulation between control (CON) and TDG-treated (TDG) groups. Band intensity was measured by ImageMaster 2-DE software version 4.95. Data are presented as the mean ± SD of volume density (%) of altered proteins in pooled samples from six rats in each group. Statistical significance between the control and TDG-treated groups was estimated by Student’s *t*-test, where *p*-values are ^†^ *p <* 0.05 and ^††^ *p <* 0.01. Full names are presented in Table 1 and abbreviation section.

**Figure 4:**
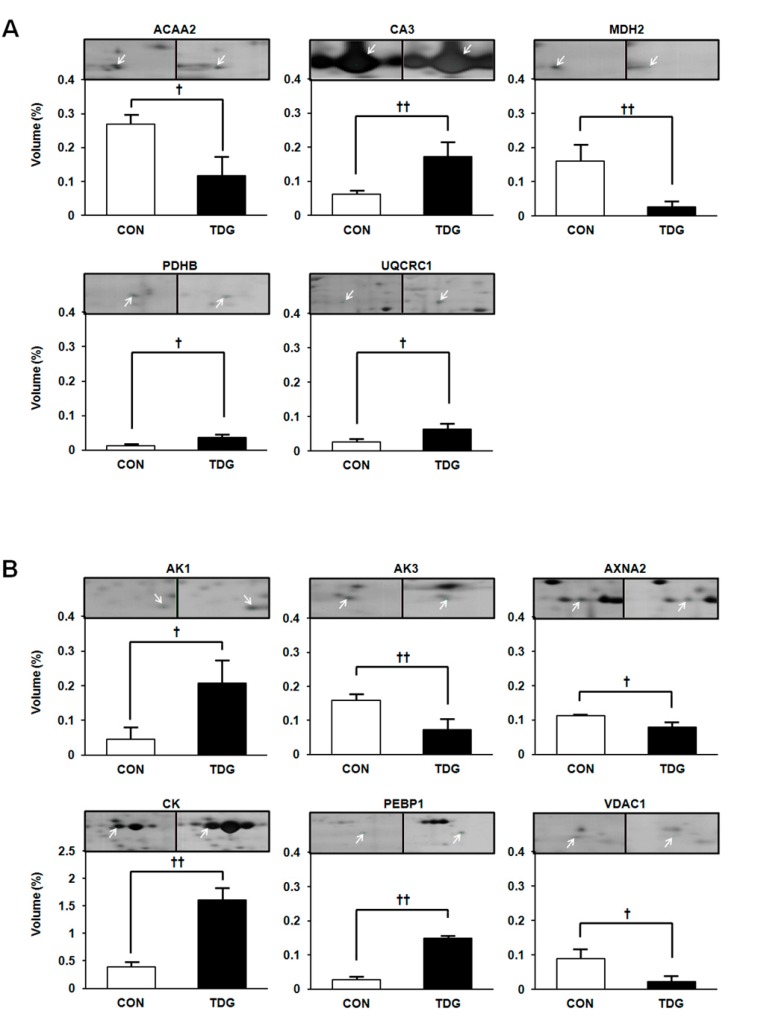
WAT proteins showing differential regulation between control and TDG-treated groups. Panel (**A**) represents proteins involved in the tricarboxylic acid (TCA) cycle; while panel (**B**) represents the signal transduction and cytoskeletal proteins. Data are presented as the mean ± SD of volume density (%) of altered proteins in pooled samples from six rats in each group. Statistical significance between the control and TDG-treated groups was estimated by Student’s *t*-test, where *p*-values are ^†^ *p <* 0.05 and ^††^ *p <* 0.01. Full names are presented in Table 1 and abbreviation section.

We divided the differentially regulated proteins between the two groups based on their functions. Group (1) includes proteins involved in carbohydrate metabolism. Seven proteins were identified in this category, including phosphoglucomutase-1 (PGM1), pyruvate kinase (PKM), glyceryldehyde-3-phosphate dehydrogenase (GAPDH), aldolase A (ALDOA), lactate dehydrogenase A chain (LDHA), triosephosphate isomerase (TPI1) and phosphoglycerate mutase 2 (PGAM2) (Figure 3). Group (2) includes proteins associated with the tricarboxylic acid (TCA) cycle. Five proteins were identified in this category, including malate dehydrogenase 2 (MDH2), pyruvate dehydrogenase E1 component subunit beta (PDHB), Cytochrome b-c1 complex subunit 1 (UQCRC1), carbonic anhydrase 3 (CA3) and acetyl-coenzyme A acyltransferase 2 (ACAA2) (Figure 4A). Group (3) includes signal transduction, and cytoskeletal proteins namely adenylate kinase isoenzyme 1 (AK1), GTP:AMP phosphotransferase (AK3), creatine kinase (CK), phosphatidylethanolamine-binding protein 1 (PEBP1), annexin A2 (ANXA2), and voltage dependent anion channel 1 (VDAC1) (Figure 4B).

### 2.4. Validation of Proteomic Data by Immunoblot Analysis

**Figure 5:**
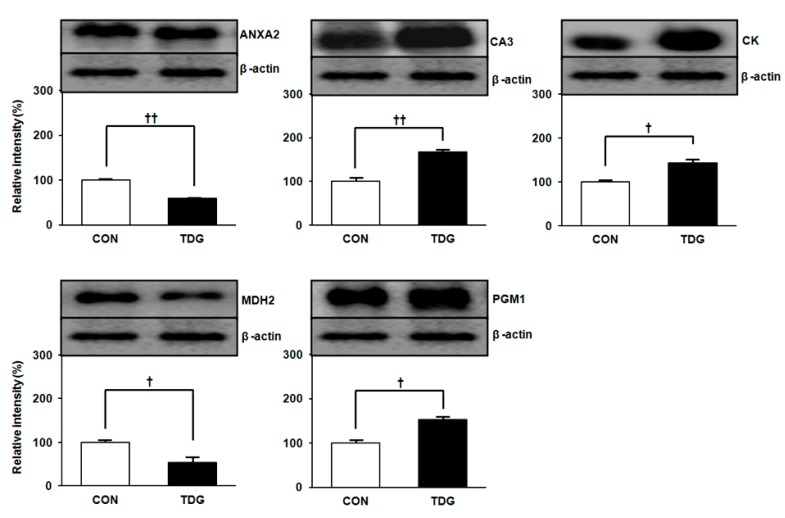
Validation of proteomic data by immunoblot analysis. Five proteins identified by 2-DE analysis were confirmed by immunoblot analysis using pooled as well as individual samples of WAT. Data are representative of three independent experiments. Image master 2-DE software version 4.95 was used to obtain the band density, and the relative intensity (%) values of proteins were normalized by β-actin levels. Statistical significance was determined by Student’s *t*-test, where *p* values are ^†^ *p <* 0.05 and ^††^ *p <* 0.01.

Proteomic data revealed a total of 32 proteins showing significant differential expression between the CON and TDG-treated groups. However, we could not rule out the possibility of technical errors and other artifacts in our proteomic data. To clarify this issue, expression levels of five WAT proteins of interest were further confirmed from individual and pooled samples by immunoblot analysis. As shown in Figure 5, regulation patterns of all proteins detected in the 2-DE protein map were exactly in line with the results of the immunoblot analysis.

### 2.5. Differential Expression of Thermogenic Marker Proteins between Control (CON) and TDG-Treated (TDG) Groups

**Figure 6:**
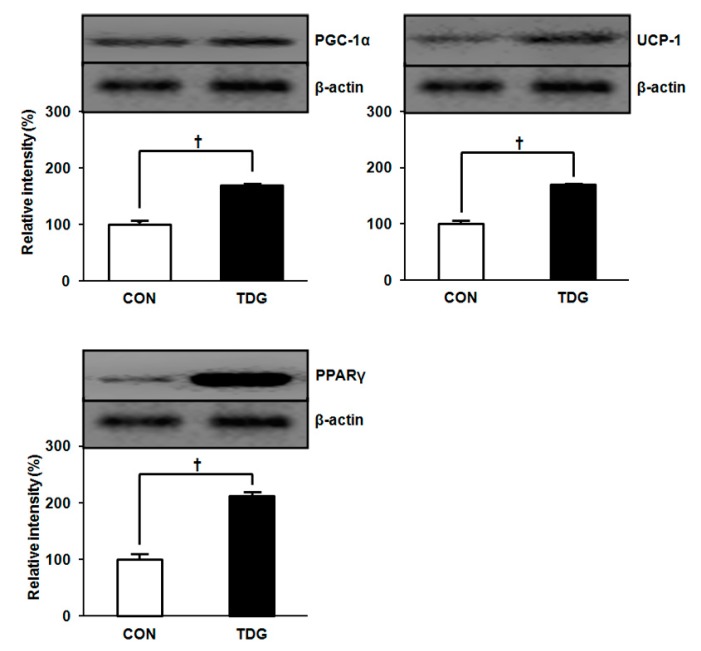
Comparison of expression patterns of thermogenic marker proteins in WAT between control and TDG-treated rats. For full names of each protein, see abbreviation section. Band intensity was estemated by ImageMaster 2-DE software. Statistical significance was determined by Student’s *t*-test, where *p* values are ^†^ *p <* 0.05.

Apart from the proteomic study, we also examined differential expression patterns of thermogenic marker proteins in WAT from each group that were not detected by proteomic analysis. Protein levels of peroxisome proliferator-activated receptor protein γ (PPARγ), uncoupling protein 1 (UCP-1) and peroxisome proliferator-activated receptor gamma coactivator1-alpha (PGC-1α) markedly increased in WAT from the TDG-treated group, suggesting a possible role for TDG in thermogenesis (Figure 6).

### 2.6. Network Analysis

**Figure 7:**
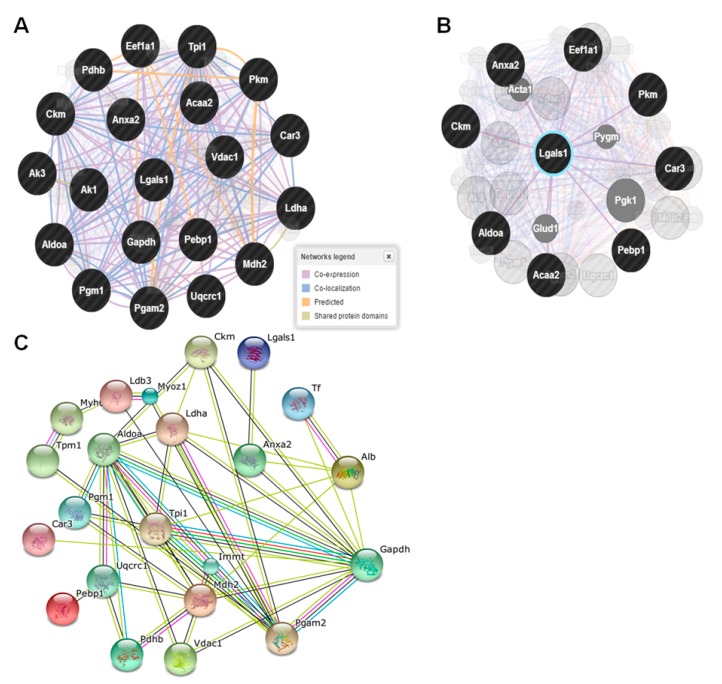
GeneMANIA and STRING, showing interaction patterns of differentially regulated proteins identified from proteomic study. Prediction results of gene interactions using GeneMANIA, which focuses on differentially expressed proteins mostly involved in metabolic and signaling pathways (in black circle), as well as a close-up screenshot of only those query genes directly linked with target protein (galectin-1, GAL1) captured with all other interactions hidden (**A**,**B**); Prediction of protein interactions between GAL1 and other differentially expressed proteins (**C**).

Most of the identified proteins were found to be related to metabolic and signaling processes when analyzed by the Protein Annotation Through Evolutionary Relationship (PANTHER) and Database for Annotation, Visualization, Integrated Discovery (DAVID) classification database. GeneMANIA was used to predict gene interactions for differentially expressed proteins upon TDG treatment. Figure 7A describes the interactions of 18 query genes, which are mostly involved in metabolic and signal transduction pathways. In addition, the STRING bioinformatics tool generated an interaction map showing direct and indirect interactions between GAL1 and differentially regulated proteins in response to TDG treatment (Figure 7B).

## 3. Discussion

**Figure 8:**
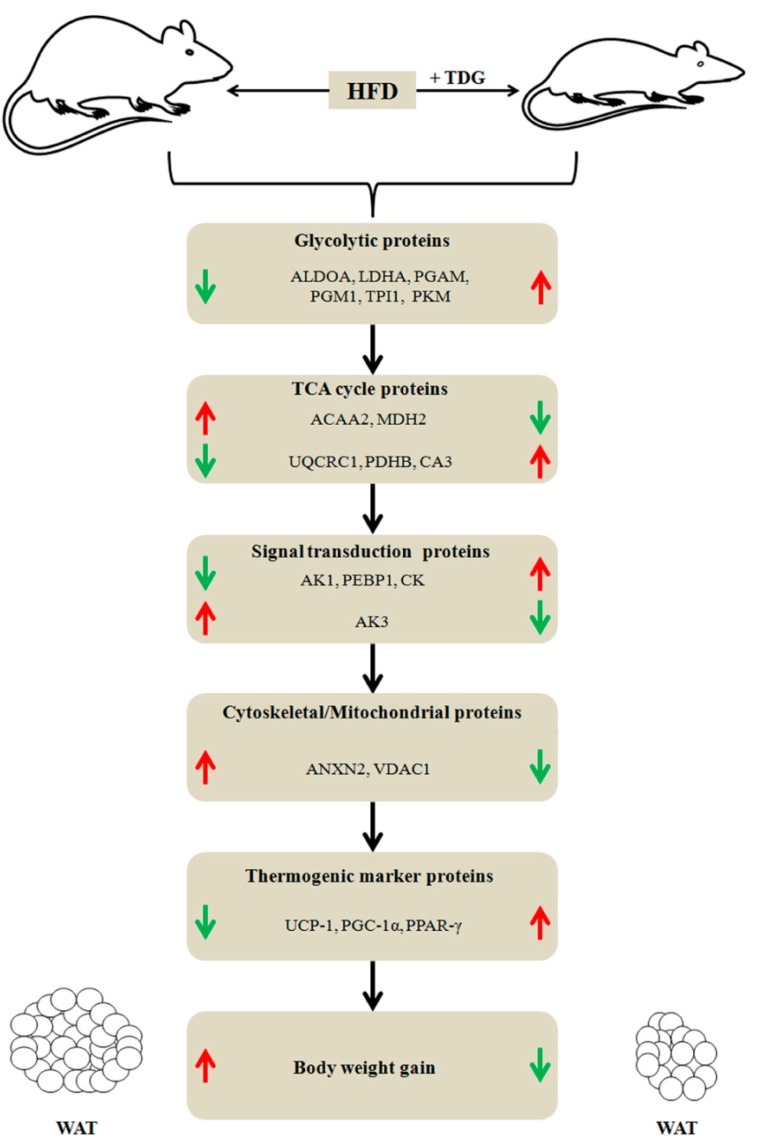
Brief summary of differentially regulated proteins, and phenotypic differences between high fat diet-control and TDG-treated rats: (↑) indicates up-regulation and (↓) indicates down-regulation of proteins. For full names of proteins, refer to Table 1 and abbreviation section.

In the present study, to identify target proteins of TDG, a potent anti-obesity drug candidate, we analyzed the WAT proteome in HFD-fed rats in response to TDG treatment. There are other known targets of thiodigalactoside, such as heat-labile enterotoxin b chain, neurocan core protein and lactose permease, but this study only focused on GAL1 as a target of TDG. As demonstrated in our recent study, GAL1 expression significantly decreases in WAT of TDG treatment group [23]. Our results provide evidence that TDG significantly decreased body weight gain and altered regulation of numerous WAT proteins (Figure 8). Although, we selected and identified 32 proteins on the basis of fold change (>1.5) and significant differential expression in response to TDG, the physiological significance of only 18 proteins is discussed below due to space limitations. Interestingly, most of the differentially expressed proteins were shown to be involved in carbohydrate metabolism. The proteins found to be involved in glycolysis include aldolase A (ALDOA), glyceraldehyde-3-phosphate dehydrogenase (GAPDH), phosphoglucomutase-1 (PGM1), pyruvate kinase (PKM), triosephosphate isomerase (TPI1), and phosphoglycerate mutase 2 (PGAM2). The overall physiological role of these glycolytic proteins is elevation of the rate of glycolysis. All six of these glycolytic proteins showed up-regulation in TDG-treated rats. An earlier study showed that increased glycolysis in hepatocytes leads to decreased body weight and adiposity [24]. Similarly, increased expression levels of glycolytic proteins in this study may have led to enhancement of glycolysis and consequently reduction of body weight gain and adiposity in TDG-treated rats.

Another interesting finding in this study is up-regulation of lactate dehydrogenase A chain (LDHA) upon TDG treatment, which is an enzyme subunit found in numerous body tissues that helps convert pyruvate into lactate, thereby generating NAD^+^ (nicotinamide adenine dinucleotide) to sustain metabolic flux [25]. Carriere and co-workers showed that lactate increases the destiny of white adipose cells in mice and humans towards a brown phenotype by altering the expression of several browning genes [26]. Expression of several proteins involved in mitochondrial activity and biogenesis is controlled by lactate [27]. The elevated level of LDHA in TDG-treated rats may explain why more pyruvate was converted into lactate, leading to reduction of body weight gain. In support of this hypothesis, we observed up-regulation of thermogenic marker proteins such as uncoupling protein-1 (UCP-1), peroxisome proliferator-activated receptor protein γ (PPARγ) and peroxisome proliferator-activated receptor gamma coactivator1-alpha (PGC-1α) by immunoblot analysis.

Another remarkable outcome of this proteomic study is the identification of differentially expressed proteins involved in the TCA cycle. Our data demonstrate decreased expression of acetyl-coenzyme A acytransferase 2 (ACAA2) upon TDG treatment. ACAA2 (also called thiolase II) is specific to thiolysis of acetoacetyl-CoA and is involved in the biogenesis of β-hydroxybutyric acid or steroid biogenesis [28]. An earlier report pointed out that β-hydroxybutyric acid inhibits adipocyte lipolysis [29]. Thus, decreased levels of ACAA2 in the TDG-treated group favored less β-hydroxybutyric acid biogenesis and may have contributed to reduction of body weight gain.

Reduced malate dehydrogenase 2 (MDH2) levels in TDG-treated rats are also an important finding in this study. MDH2 is abundantly found in the mitochondrial matrix as a key enzyme in the citric acid cycle and catalyzes the oxidation of malate to oxaloacetate, thereby activating adenosine monophosphate (AMP) activated protein kinase [30]. Several studies have reported that AMP-activated protein kinase has an anti-lipolytic effect [31,32]. Thus, decreased levels of MDH2 are likely to be positively correlated with body weight loss.

We detected that TDG up-regulated cytochrome b-c1 complex subunit 1 (UQCRC1) in response to TDG treatment, which is a part of the respiratory chain protein cytochrome b-c1 complex or complex III involved in biological energy conversion [33]. UQCRC1 reversibly oxidizes hydroquinone and reduces cytochrome c, the two major redox pools of electron transport chain in mitochondria [34]. Recent reports have studied the activity and abundance of UQCRC1 in the context of different metabolic diseases. For example, Hastie *et al.* demonstrated that women with obesity or diabetes show significantly decreased protein levels of placental UQCRC1 [35]. UQCRC1 expression was also found to be reduced in visceral adipose tissues from obesity-prone mice [36]. Our proteomic data also support these findings, suggesting that reduction of protein levels of UQCRC1 in HFD-fed rats may have detrimental consequences on WAT function.

Pyruvate dehydrogenase E1 component subunit beta (PDHB) is the main enzymatic component of the pyruvate dehydrogenase complex (PDC), which catalyzes conversion of pyruvate to acetyl-CoA and CO_2_, thereby linking the glycolytic pathway to the TCA cycle. Liver-specific PDC deficiency may lead to enhancement of lipogenesis and peripheral insulin sensitivity in mice [37]. Here, we observed increased expression of PDHB in WAT from TDG-treated rats for the first time. Thus, there may be a link between PDHB elevation and decreased lipogenesis.

Elevated expression of carbonic anhydrase 3 (CA3) in TDG treated-rats is another intriguing finding of the current study. CA3 is a member of the multi-enzyme family that catalyzes reversible hydration of carbon dioxide and is abundantly found in adipocytes and the liver [38]. CA3 activity and concentration are reduced in WAT from obese animals [39]. Our results support the idea that TDG may act as an inducer of CA3 via binding with GAL1, leading to decreased body weight gain. However, further studies are required to directly test this hypothesis.

Apart from metabolic proteins, we also observed proteins involved in signal transduction. Unexpectedly, we detected up- and down-regulation of AK1 and AK3 in TDG-treated rats, respectively. AK1 and AK3 are ubiquitous enzymes that contribute to the homeostasis of cellular adenine and guanine nucleotide pools and cell energy economy through regulation of nucleotide ratios in different intracellular compartments [40,41]. These are also nuclear-encoded proteins and synthesized in the cytoplasm. AK1 is located in the cytosol, whereas AK3 is located in mitochondrial matrix and uses GTP instead of ATP as a phosphate donor [42]. These enzymes allow the utilization of the second high energy bond of β-phosphoryl in ATP, thereby doubling energetic potential. They also play important functions in tissues with high energy demands as well as those under metabolic stress [41,43]. However, AK1 and AK3 show opposite regulation patterns compared to our findings in obese mice [22,44]. Thus, further studies are needed to prove whether elevated AK1 and decreased AK3 contribute to reduction of body weight gain.

One of the most striking results in this study is the up-regulation of phosphatidylethanolamine-binding protein 1 (PEBP1) in TDG-treated rats. PEBP1 is also called Raf kinase inhibitory protein (RKIP) and belongs to an evolutionary conserved family of PEBPs, playing an important function as an inhibitor of kinase signaling pathways and metastasis [45]. PEBP1 can interrupt the Ras-Raf-MEK-ERK and JNK pathways by breaking interactions between Raf-1 and its MEK substrate. RKIP has also been found to be a suppressor of metastasis [46]. The JNK pathway is involved in the development of obesity and diabetes [47,48]. JNK knockout HFD-fed mice show improved insulin sensitivity and reduced adiposity in contrast to wild-type [49]. Thus, our proteomic data support these findings, suggesting that RKIP might play a role in the prevention of obesity.

For the first time, we have observed significant elevation of creatine kinase (CK) in WAT from TDG-treated rats. One of the main functions of CK is the regulation of oxidative phosphorylation and energy transport by catalyzing reversible conversion of creatine and ATP into ADP and phosphocreatine [50]. However, there are numerous conflicting reports regarding the abundance and activity of CK in obese as well as diabetic patients [44,51]. The protein levels of CK have been shown to be 30% higher in obese/overweight women compared to lean counterparts [44]. Up-regulation of CK has recently been shown to safeguard mice from heart failure, which is well known to be associated with obesity and weight gain [51]. Here, it is difficult to interpret the increase in CK protein levels in the TDG-treated group showing weight loss, but we can postulate that higher expression of CK in response to TDG treatment may regulate body mass by maintaining homeostasis of ATP synthesis and consumption.

Another notable result of this study is the reduced expression of annexin A2 (ANXA2) and voltage-dependent anion channel 1 (VDAC1) in TDG-treated rats. Earlier reports demonstrated that ANXA2 is essential for endocytosis, exocytosis, lipid raft formation, and signal transduction through CD44 cell receptor interactions [52,53]. ANXA2 and prohibitin form a complex receptor in lipid rafts of WAT that plays an important function in WAT development and lipid accumulation during adipocyte differentiation [54]. Here, we hypothesize that reduction of ANXA2 levels may inhibit body weight gain by decreasing lipogenesis.

VDAC1 is a porin ion channel located mostly on the outer mitochondrial membrane that forms a voltage-dependent anion-selective channel, allowing the diffusion of small specific hydrophilic molecules [55]. VDAC1 forms the main interface between mitochondrial and cellular metabolic processes [56]. Elevated levels of hydrophilic molecules have been found in inguinal WAT from obese Zucker rats [57]. Interestingly, we observed decreased levels of VDAC1 in TDG-treated lean subjects, which may explain the decreased entry of hydrophilic molecules and subsequent weight loss. Thus, our results may provide insight into the potential of VDAC1 as a rational target for therapeutics.

Surprisingly, ANXA2, CA3, ACAA2, PKM, ALDOA, PEBP1, and CK were identified as partner proteins in the context of lipogenesis, adipocyte signaling, adipocyte fat accumulation, and cytoskeletal dynamics. The GAL1 interactome map generated by GeneMANIA confirmed a direct link between GAL1 and all abovementioned seven partner proteins.

In conclusion, the present proteomic study revealed that TDG, a potent inhibitor of GAL1, shows promising anti-obesity effects through alteration of WAT protein expression. Further, we notably observed significant changes in expression patterns of CA3, VDAC1, PEBP1, ANXA2, and LDAC upon TDG treatment. Accordingly, these results suggest that TDG may effectively prevent obesity, and TDG-responsive proteins could be used as novel targets for obesity treatment.

## 4. Experimental Section

### 4.1. Animal Experiments

Five-week-old Sprague-Dawley (SD) rats were purchased from Daehan Experiment Animals (Seoul, Korea), and rats were kept separately in each cage maintained at a temperature of 24 ± 2 °C and relative humidity of 30%~70%. All animals were provided with water and normal chow *ad libitum* for 1 week for acclimation to a new environment. After adaption, rats were randomly divided into two groups, *viz.* HFD control (CON) and TDG-treated HFD (TDG) groups. PBS and TDG dissolved in PBS were injected peritoneally into the CON and TDG groups, respectively, at a dose of 5 mg/kg body weight once per week for 35 days. HFD contains 45% fat as an energy source (Korea Lab., Hanam, Korea), and the dietary makeup used in this study is presented in Table 2. Free access to food and water was provided to rats during the experimental period. Food intake and body weight were measured weekly. Food efficiency was calculated by total difference in body weight/total food consumption. After the treatment for 5 weeks, six rats from each group were separated and euthanized by cervical dislocation. Animal experiments were performed (under project identification code, DUIACC-2014-3-0321-001) with the consent of the Advisory Committee for Laboratory Animal Care and Use of Daegu University. All events were executed in accordance with the Guide for the Care and Use of Laboratory Animals issued by the National Institutes of Health (NIH).

### 4.2. Preparation of WAT Samples for 2-DE

After animal euthanization, abdominal WAT was separated from all rats, after which tissues were washed with cold saline solution. WAT was pulverized under liquid nitrogen into fine powder and stored at −80 °C until further use. About 40 mg of pulverized tissues was lysed in 200 μL of rehydration buffer containing 7 M urea, 2 M thiourea, 20 mM DTT, 1 mM phenylmethylsulfonyl fluoride (PMSF), 4% CHAPS (3-[(3-cholamidopropyl)dimethylammonio]-1-propanesulfonate), 2% immobilized pH gradient (IPG) buffer (Ampholyte 3/10, Bio-Rad, Hercules, CA, USA), and bromophenol blue traces (Sigma-Aldrich, St. Louis, MO, USA). During the sample preparation events, pulverized tissue samples were kept on ice, and the homogenizer (PT 1200E, Kinematica, Luzern, Switzerland) was operated at 3 × 45 s with a gap of 1 min on ice between each round. The solublized samples were vortexed for 1 min three times and then centrifuged at 13,000× *g* for 20 min at 4 °C, followed by trichloroacetic acid precipitation as mentioned in our previous paper [21]. After the protein pellet was dried, it was resuspended in 200 μL of rehydration buffer and stored in −80 °C until protein analysis. Protein quantification was carried out using the Bradford method. In order to increase the reagent compatibility and to avoid detergent interference, the sample was highly diluted (10 μL solubilized sample + 790 μL double distilled water + 200 μL Bio-Rad protein reagent concentrate) and Bovine serum albumin (Generay Biotech, Shanghai, China) standards were also dissolved in the same rehydration buffer. And, we also use the RC/DC protein assay (Bio-Rad) to make sure the protein concentration determination in the presence of reducing agents and detergents.

**Table 2:** Compositions of normal diet (ND) and high fat diet (HFD).

Ingredient	ND Composition by Weight, g/kg	HFD
Casein	200	265
Sucrose	100	90
Corn starch	397.4	0
Maltodextrin	132	160
Cellulose	50	65.5
Soybean oil	70	30
Lard	0	310
Mineral mix	35	48
Calcium phosphate, dibasic	0	3.4
Vitamin mix	10	21
l-cystine	3	4
Choline bitartrate	2.5	3
Total (kcal/kg)	3800	5100

### 4.3. 2-DE

2-DE analysis was performed using pooled protein extracts of WAT from six rats per group. 2-DE experiments were conducted using the optimized methods outlined in our previous reports [20,21]. Pooling of WAT protein extracts of six rats from each group was performed to avoid gel-to-gel variations, and 2-DE of each group was repeated three times. Briefly, immobilized pH gradient (IPG)-isoelectric focusing (IEF) of WAT protein samples was carried out on pH 3–10 and 18 cm IPG immobilized DryStrips (GE Healthcare, Buckinghamshire, UK) in a protein IEF cell (Bio-Rad) using the protocol recommended by the manufacturer. IPG strips were rehydrated passively for 24 h in strip holders with 350 μL of rehydration buffer, including 7 M urea, 2 M thiourea, 4% CHAPS, 1 mM PMSF, 20 mM DTT, 2% IPG buffer, and 100 μg of WAT protein. IEF was carried out as follows: 15 min at 250 V, 3 h at 250–10,000 V, and then held at 500 V until the second dimension was run. Strips containing focused proteins were thawed, equilibrated, and reduced in equilibration solution (6 M urea, 2% SDS, 1% DTT, 30% glycerol, and 50 mM Tris-HCl at pH 6.8) for 20 min, followed by alkylation in the same buffer, except for replacement of DTT with 2.5% iodoacetamide (Bio-Rad) for 20 min. Equilibrated strips were subjected to 20 × 20 cm 12% sodium dodecyl sulfate polyacrylamide gel electrophoresis (SDS-PAGE) for resolution in the second dimension. After this, fractionation was carried out with the Laemmlia SDS-discontinuous system at a constant voltage of 15–20 mA per gel. For image analysis and peptide mass fingerprinting (PMF), a total of six gels, including three gels per group with separated proteins, were visualized by silver staining as outlined previously [20].

### 4.4. Image Acquisition and Data Analysis

A UMAX Power Look 1120 (Maxium Technologies, Akron, OH, USA) scanner was used to capture and store images of 2-DE gels. ImageMaster 2-DE software V4.95 (GE Healthcare) was used to evaluate images according to the manufacturer’s instructions and methods outlined in our previous studies [58,59]. A reference gel was selected from three gels of the control group, and detected spots from the TDG-treated group were matched with those in the reference gel. Calculation of relative optical density and relative volume was carried out in order to correct the differences in gel staining. Each spot intensity volume was processed by background subtraction and total spot volume normalization, and the resulting spot volume percentage was used for comparison.

### 4.5. Protein Identification

PMF was used for protein identification, in which protein spots were excised from silver-stained 2-DE gels using a spot cutter and digested with trypsin (Promega, Madison, WI, USA), mixed with α-cyano-4-hydroxycinnamic acid (CHCA) in 50% acetonitrile (ACN)/0.1% trifluoroacetic acid (TFA) and subjected to MALDI-TOF-MS analysis (Microflex LRF 20, Bruker Daltonics, Billerica, MA, USA). Spectra were collected from 350 shots per spectrum over a *m*/*z* range of 600–3000, and internal calibration was automatically performed as two-point-calibration using trypsin auto-digestion peaks (*m*/*z* = 842.5099, 2211.1046). A peak list was generated using Flex Analysis (ver. 3.0) (Bruker Daltonics, Billerica, MA, USA), and the threshold used for picking the peaks was as follows: 5000 for minimum resolution of monoisotopic mass, 5.0 for signal-to-noise (S/N) threshold. Mass of peptides was matched with the theoretical peptides of all proteins available in the NCBI database using MASCOT developed by Matrixscience (Bruker Daltonics, Boston, MA, USA). For database search, the following parameters were used: trypsin as the cleaving enzyme, a maximum of one missed cleavage, iodoacetamide (Cys) as a complete modification, oxidation (Met) as a defined modification, monoisotopic masses, and a mass tolerance of ±0.1 Da. Protein score is −10 × log (*p*), where *p* is the probability that the observed match is a random event, and protein scores greater than 61 are significant (*p <* 0.05).

### 4.6. Immunoblot Analysis

For confirmation of differentially regulated proteins identified from the 2-DE protein map, Western blot analysis was performed. Tissue lysates of six rats from each group were prepared by using RIPA buffer (Sigma-Aldrich), homogenized, and centrifuged at 12,000× *g* for 20 min. Upper lipid layer was discarded and lower liquid collected, after which protein content was quantified by the Bradford method. Extract was diluted in sample buffer (50 mM Tris at pH 6.8, 2% SDS, 10% glycerol, 0.1% bromophenol blue, and 5% β-mercaptoethanol) and heated for 3–5 min in a boiling water bath. Samples were then subjected to 10%–12% SDS-PAGE and transferred to PolyScreen membranes (New England Nuclear, Boston, MA, USA). Membranes were subsequently blocked for a minimum of 1 h using 5% non-fat dry milk or BSA in TBS (10 mM Tris-HCl, 150 mM NaCl, pH 7.5) containing 0.1% Tween-20 (TBS-T). The membrane was then rinsed three times with TBS-T buffer, followed by incubation for 2–4 h with 1:1000 dilution of primary antibody. The following antibodies were used in this study: anti-goat UCP-1, MDH2, PGM1, PPARγ, and CA3; anti-mouse CK and β-actin (Santa Cruz Biotechnology, Santa Cruz, CA, USA); and anti-rabbit ANXA2 (Cell Signaling Technology, Beverly, MA, USA) and PGC-1α (Santa Cruz Biotechnology). After antibody incubation, washing was carried out three times by TBS-T, followed by incubation with horseradish peroxidase-conjugated anti-rabbit immunoglobulin G (IgG), anti-goat IgG, and anti-mouse IgG secondary antibody (1:1000; Santa Cruz Biotechnology) for 1 h and then visualization by enhanced chemiluminescence (iNtRON Biotechnology, Seoul, Korea). Image acquisition was carried out by scanning with UMAX PowerLook 1120 (Maxium Technologies) and digitalization using image analysis software (KODAK 1D, Eastman Kodak, Rochester, NY, USA).

### 4.7. Network Analysis

For gene/protein classification, identified proteins obtained from proteomic data were analyzed using the Protein Annotation through Evolutionary Relationship (PANTHER) and Database for Annotation, Visualization, Integrated Discovery (DAVID) classification systems. These public databases make it possible to systematically divide large protein lists in an attempt to assemble a summary of related biological functions, pathways, and protein classes. Finally, predicted gene and protein interactions between GAL1 and its interacting partner proteins identified in this study were collected by using two different predictive web interfaces, GeneMANIA and STRING. These interfaces generate a list of genes and proteins with functional similarity based on available genomics and proteomics databases. Protein–protein interactions among the identified proteins were analyzed by using STRING (Search Tool for the Retrieval of Interacting Genes) ver. 9 (http://string.embl.de) based on the following analysis parameters; Rattus norvegicus, and confidence level 0.4. STRING is a freely available database, based on known and predicted protein interactions, that quantitatively integrates interaction data from high throughput experiments, genomic context, and co-expression. GeneMANIA is a flexible user-friendly web interface for predicting hypotheses about gene function, analyzing gene lists, and arranging genes for functional assay.

### 4.8. Statistical Analysis

All the results are expressed as the mean ± SD. Statistical significance between control and TDG-treated groups were calculated by using Student’s *t-*test, wherein level of significance was set at either *p <* 0.01 or *p <* 0.05.

## Abbreviations

2-DE: two-dimensional gel electrophoresis
AK1: adenylate kinase isoenzyme 1
AK3: adenylate kinase isoenzyme 3
GAPDH: glyceryldehyde-3-phosphate dehydrogenase
PGM1: phosphoglucomutase-1
PGAM2: phosphoglycerate mutase 2
ALDOA: aldolase A
TPI1: triosephosphate isomerase
ANXA2: annexin A2
UCP-1: uncoupling protein 1
PPARγ: peroxisome proliferator-activated receptor protein γ
PGC-1α: peroxisome proliferator-activated receptor gamma coactivator 1-alpha
LDHA: l-lactate dehydrogenase A chain
UQCRC1: Cytochrome b-c1 complex subunit 1
RKIP: Raf kinase inhibitory protein
CA3: carbonic anhydrase 3
VDAC1: voltage-dependent anion channel
PEBP1: phosphatidylethanolamine-binding protein 1
LDAC: lactate dehydrogenase A chain
TCA: tricarboxylic acid
PMF: peptide mass fingerprinting
CHCA: α-cyano-4-hydroxycinnamic acid
TFA: trifluoroacetic acid
